# Role of Different Antithrombotic Regimens after Percutaneous Left Atrial Appendage Occlusion: A Large Single Center Experience

**DOI:** 10.3390/jcm10091959

**Published:** 2021-05-02

**Authors:** Patrizio Mazzone, Alessandra Laricchia, Giuseppe D’Angelo, Giulio Falasconi, Luigi Pannone, Luca Rosario Limite, David Zweiker, Damiano Regazzoli, Andrea Radinovic, Alessandra Marzi, Eustachio Agricola, Luigia Brugliera, Antonio Colombo, Paolo Della Bella, Matteo Montorfano

**Affiliations:** 1 Department of Arrhythmology and Cardiac Electrophysiology, IRCCS San Raffaele Scientific Institute, 20132 Milan, Italy; mazzone.patrizio@hsr.it (P.M.); giuliofalasconi@gmail.com (G.F.); pannone.luigi@hsr.it (L.P.); limite.luca@hsr.it (L.R.L.); davidzweiker@gmail.com (D.Z.); radinovic.andrea@hsr.it (A.R.); marzi.alessandra@hsr.it (A.M.); dellabella.paolo@hsr.it (P.D.B.); 2 Interventional Cardiology Unit, Cardiology and Cardiothoracic Surgery Department, San Raffaele University Hospital, 20132 Milan, Italy; laricchia.a@gmail.com (A.L.); montorfano.matteo@hsr.it (M.M.); 3 Interventional Cardiology Unit, Humanitas Research Hospital, 20132 Milan, Italy; damiano.regazzoli@gmail.com (D.R.); ac54344@gmail.com (A.C.); 4 Noninvasive Cardiology Unit, Cardiology and Cardiothoracic Surgery Department, San Raffaele University Hospital, 20132 Milan, Italy; agricola.eustachio@hsr.it; 5 Department of Rehabilitation and Functional Recovery, I.R.C.C.S. San Raffaele Scientific Institute, Vita-Salute University, 20132 Milan, Italy; brugliera.luigia@hsr.it

**Keywords:** anticoagulant therapy, antithrombotic therapy, left atrial appendage occlusion, stroke

## Abstract

Background: Optimal antithrombotic therapy after left atrial appendage (LAA) occlusion is still not clear. The aim of this study was to investigate the role of different antithrombotic regimens after the procedure. Methods and Results: We retrospectively analyzed data of 260 patients who underwent LAA occlusion and divided them into four groups according to therapy at discharge: dual antiplatelet therapy (group A, 71.5%); oral anticoagulants (group B, 19%); “minimal” antithrombotic therapy (single antiplatelet agent or without any antithrombotic therapy; group C, 4.5%) and other therapeutic regimens (such as a combination of antiplatelets and anticoagulants; group D, 4.5%). We analyzed baseline characteristics, procedural data, and clinical and transesophageal follow-up for each group. The incidence of adverse events was low in the whole population and had a similar distribution among groups. The majority of bleeding events was registered during the first 3 months after the procedure (34 out of 46, 70%). Ischemic events (2%), as well as silent left atrial thrombosis, were rare and not significantly higher in the population discharged with “minimal” antithrombotic therapy. Conclusion: Our experience seems to suggest that LAA occlusion was associated with a low incidence of adverse events, regardless of antithrombotic therapy. A “minimal” drug regimen may be feasible without losing efficacy on embolic prevention for patients with high bleeding risk.

## 1. Introduction

Atrial fibrillation (AF) is a common, age-related arrhythmia, with 1.5–2% prevalence in the general population. It is associated to a fivefold increased risk of embolic strokes, which usually determine significant morbidity and mortality [1,2].

Oral anticoagulation (OAC), both with vitamin K antagonist (VKA) and with new oral anticoagulant (NOACs), significantly decreases the risk of stroke [3,4,5,6], while increasing the incidence of bleeding events. Percutaneous left atrial appendage (LAA) occlusion has emerged as an alternative to anticoagulant therapy in patients with high bleeding risk [7,8,9], leading to an increased marketing of many devices in Europe in the last five years [10,11,12].

However, the optimal medical therapy after LAA occlusion is still an unresolved issue and varies by type of LAA occluder. Usually, some type of intensive antithrombotic therapy is prescribed for a period of at least 1–3 months and until device endothelialization, followed by a de-escalation strategy, which consists of single antiplatelet therapy in the majority of cases. This strategy is based on the results of previous large randomized clinical trials that enrolled patients without any contraindication to oral anticoagulation, or on the experience with other percutaneously implanted devices as interatrial septum occluders. As the “real world” population undergoing LAA occlusion is highly heterogeneous and characterized by variable bleeding risk, this standard approach is not consistently applied and patients are discharged with different therapeutic regimens according to clinician choice.

The aim of this study is to investigate the role of antithrombotic therapy after LAA occlusion in a high-volume center, with particular interest in the incidence of ischemic and hemorrhagic complications during the first three months after discharge.

## 2. Methods

According to Hospital Internal Ethics Committee policy, we retrospectively collected data of consecutive patients who underwent LAA occlusion between August 2010 and October 2016 in a single high-volume center in Milan, Italy. All participants had previously provided informed consent for future data collection and analysis. Patients with valvular AF, mechanical heart valves, different absolute indications to OAC (e.g., pulmonary embolism), need of concomitant procedures (e.g., percutaneous mitral valve repair or transcatheter aortic valve replacement) and active oncologic disease were excluded from the analysis.

The procedure was performed according to national and international recommendations, as well as the center’s standard operating protocol. The decision to perform LAA occlusion was left to the attending physician. At admission, the patient’s detailed history including risk scores for bleeding and thromboembolism (e.g., CHA_2_DS_2_-VASc and HAS-BLED) were evaluated. At baseline, transesophageal echocardiography (TEE) was performed in all patients to exclude a LAA thrombus and to evaluate the feasibility of the procedure. Baseline computed tomography (CT) scan was considered optional.

LAA occlusion was performed under general anesthesia and continuous monitoring with TEE and fluoroscopy, with systemic heparinization targeting an activated coagulation time over 250 s. The type of device was selected at the operators’ discretion and market availability. Utilized devices included the Watchman and second-generation Watchman FLX (Boston Scientific, Natick, Massachusetts, USA), and Amplatzer Cardiac Plug (ACP) and the newer Amulet device (Abbott—SJM, Santa Clara, California, USA). Before discharge, chest X-ray, and transthoracic echocardiography were performed to exclude acute complications.

Post-discharge antithrombotic therapy was tailored on the basis of the individual thromboembolic and bleeding risk. The following four principles were adhered:

(1). Standard strategy was dual antiplatelet therapy (DAPT) including acetylsalicylic acid (ASS) 81-300 mg daily and clopidogrel 75 mg daily, without the administration of a loading dose. After three months, step down to single antiplatelet therapy (SAPT; usually ASS 81-300 mg daily) was recommended if follow up TEE showed absence of major leaks or device-related thrombosis. In this analysis, all patients on DAPT at discharge were summarized into group A.
(2). In case of higher embolic than hemorrhagic risk (such as repeated ischemic events) and the absence of contraindications to OAC therapy, the preferred strategy was oral anticoagulation with VKA or NOACs at discharge, followed by de-escalation therapy (to DAPT or SAPT) after 3 months. Those patients were stratified into group B.
(3). In case of prohibitive hemorrhagic risk (such as recent major bleeding), patients were discharged on SAPT or without any antithrombotic medication (group C)
(4). A minor number of patients with an indication for both anticoagulant and antiplatelet therapy (mainly those with concomitantly treated coronary artery disease), was discharged on triple antithrombotic therapy (including OAC, ASS, and clopidogrel) or on the combination of OAC and clopidogrel. Patients with combination of OAC with ASS, clopidogrel or both were summarized into group D.

All patients were clinically evaluated after 3 months, by telephone contact or preferably by clinical visit at our center. Transesophageal echocardiography was performed during the same outpatient visit, in absence of contraindications.

Procedural success was defined as successful delivery and immediate evidence of correct position of the device and complete exclusion of LAA, as assessed by intra-procedural imaging. According to indications from Valve Academy Research Consortium (VARC), adverse events both on short and on long-term follow-up (FU) were evaluated [13].

Primary endpoint was a composite of all-cause mortality, ischemic stroke, transient ischemic attack (TIA), intracranial bleeding, major bleeding, minor bleeding, or asymptomatic device thrombosis at transesophageal FU.

Secondary endpoints were all-cause mortality and cardiovascular mortality, ischemic and hemorrhagic stroke, transient ischemic attack, major bleedings (overt bleeding associated with a drop in the hemoglobin level of at least 3.0 g/dl or requiring transfusion) and minor bleedings (any bleeding that does not qualify as life-threatening, disabling or major). TEE-guided secondary endpoints at three months were evidence of peri-device leaks (defined as flow diameter ≥ 3 mm) or thrombosis (on the device and in the left atrium).

Statistical analysis was performed with SPSS 21.0 software (SPSS Inc., Chicago, Illinois). Continuous variables are expressed as mean (and SD) and were tested with the one-way ANOVA and Kruskal–Wallis tests if normally or non-normally distributed respectively. Categorical variables are expressed as absolute numbers (and percentages) and were tested with the chi-square test or Fisher’s exact test. A two-sided *p* value < 0.05 was considered statistically significant.

Time to event analysis was performed using Kaplan–Meier estimates with log-rank test used for comparisons among groups.

## 3. Results

We retrospectively analyzed data of 300 consecutive patients who underwent LAA closure. The final analyzed database consisted of 260 patients, since 40 patients met exclusion criteria (Table S1).

Out of all patients, 71.5% (*n* = 186) received dual antiplatelet therapy (Group A), 19.0% (*n* = 50) were on OAC treatment (Group B), and 4.5% (*n* = 12) on a “minimal” antithrombotic therapy (Group C). Remaining patients (4.5%, *n* = 12) were discharged on different therapeutic regimens (Group D; one patient with “triple” therapy, remaining with one antiplatelet plus one anticoagulant agent).

Baseline characteristics are reported in Table 1; mean age of the overall population was 73 ± 9 years, 67% were men with no significant differences between groups.

The overall population had both high thrombotic and hemorrhagic risk (mean CHA_2_DS_2_-VASc 3.8 ± 1.7, mean HAS-BLED 3.6 ± 1.4); patients discharged on “minimal” antithrombotic therapy had an even worse risk profile (as expressed by even higher ischemic and hemorrhagic risk scores). Moreover, left-ventricular ejection fraction was lowest in group B and coronary artery disease was most prevalent in group D.

**Figure 1:**
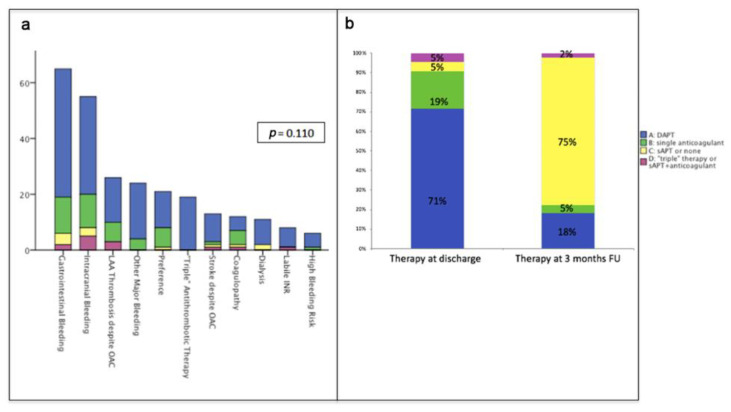
Figure (**a**) describes distribution of therapies at discharge based on indications to left atrial appendage (LAA) occlusion. Figure (**b**) shows therapies at discharge and after change after first follow-up evaluation (74 days, IQR 50-107). OAC = oral anticoagulation; INR = international normalized ratio; DAPT: double antiplatelet therapy; sAPT: single antiplatelet therapy.

Indications to LAA occlusion are reported in Figure 1a: they range from previous bleedings (mainly gastrointestinal bleedings or intracranial hemorrhage) to failure of anticoagulant therapy (ischemic stroke or demonstrated LAA thrombus despite OAC) to patient preference. While there was no significant association between indication categories and groups, we observed a trend towards preferred administration of DAPT in patients with a history of major bleeding or with labile INR. Interestingly, some patients were treated with LAA occlusion in order to avoid co-administration of double antiplatelet therapy and anticoagulant therapy.

Procedural data are reported in Table 2. A total of 82 patients (32%) were implanted with a Watchman device, 5 (2%) with a Watchman FLX, 55 (21%) an Amplatzer Cardiac Plug, and 118 (45%) an Amplatzer Amulet. Technical success was reached in all patients (100%). In 11 procedures, the device was repositioned and in 5 cases device size was changed during the procedure.

Clinical follow up was available for 253 patients (97.3%) with a median of 420 days (IQR 250-704). Transesophageal evaluation at 3 months (performed after 74 days, IQR 50-107) was available in 74.6% of cases. As shown in Figure 1b, therapy was then modified in over 70% of patients, usually with a de-escalation strategy.

**Figure 2:**
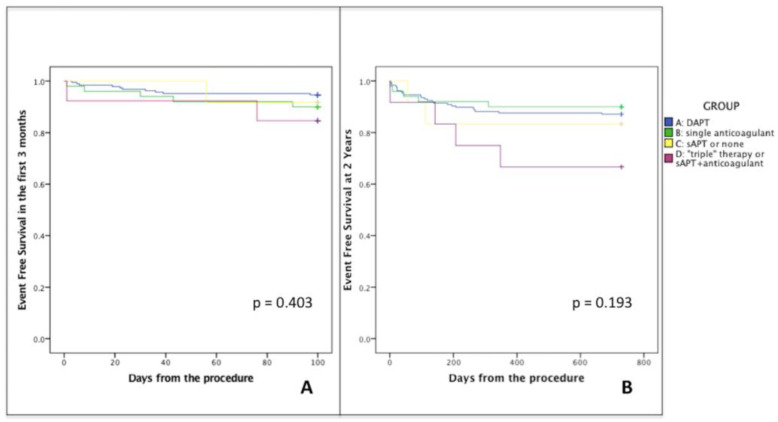
Kaplan–Meier for event free survival at 3-months (**A**) and 2-year (**B**) FU for each group: composite endpoint of left atrial thrombus on transesophageal echocardiography, ischemic stroke, transient ischemic attack, intracranial bleeding, major and minor bleeding, and all-cause mortality. GROUP A dual antiplatelet therapy; GROUP B anticoagulant therapy; GROUP C single antiplatelet therapy or no therapy; GROUP D combination of antiplatelet and anticoagulant therapy.

Table 3 and Figure 2A summarize 3 months follow up data; notably, no patient experienced more than one adverse event at follow-up and a similar distribution of events was observed among groups.

Event free survival at 2 years of FU for each group was not different between groups (*p* = 0.193; Figure 2B, Table 4).

## 4. Discussion

The main findings of our research were:

1. The incidence of both hemorrhagic and ischemic events after percutaneous LAA closure was low, irrespective to chosen antithrombotic therapy. The predicted high risk (mean CHA_2_DS_2_-VASc 3.8 ± 1.7, mean HAS-BLED 3.6 ± 1.4) did not reflect in a high incidence of bleedings or embolic events.
2. The majority of hemorrhagic events in our population occurred early after the procedure; actually, over 70% of bleedings happened in the first three months.
3. Moreover, we observed a low rate of adverse events in the group discharged with single antiplatelet or without any therapy, and those were only bleeding events.

These data, although resulting from an observational study, underline the importance of choosing the appropriate antithrombotic therapy after LAA occlusion. As LAA is considered the main source of thrombus embolization in AF patients, its occlusion is an attractive non-pharmacological alternative for stroke prophylaxis in non-valvular AF patients at high ischemic risk [14]. However, an increased bleeding risk persists in the first 45–60 days after the procedure when an antithrombotic therapy is generally deemed necessary during device endothelialization.

Therefore, in this complex scenario involving heterogeneous patients and devices, optimal medical therapy after the implantation of a LAA occluding device is still an unresolved issue. First of all, we lack randomized trials directly comparing different medical regimens; secondly, most observational studies are centered on a single device; finally, most patients with absolute contraindications to anticoagulation have a high hemorrhagic risk that prevent them to be safely treated with antiplatelet drugs or other antithrombotic therapies, at least in the context of a randomized clinical trial.

Since ischemic and hemorrhagic risks (reflected by CHA_2_DS_2_-VASc and HAS-BLED scoring systems) share many risk factors, the post-procedural antithrombotic strategy may be tailored for the patient, also taking into account other patient-related risk factors. In this analysis, there were significant differences in the bleeding risk (as calculated with the HAS-BLED score) between all groups. Differences in other comorbidities (such as coronary artery disease and reduced left-ventricular ejection fraction) between groups underline the fact that also other factors were considered when choosing the right discharge antithrombotic medication. This resulted in four very heterogeneous groups with differences in baseline bleeding and thromboembolic risk.

In the only randomized trials on this topic (PROTECT-AF and PREVAIL) patients received warfarin and warfarin plus ASS, respectively, for 45 days, followed by DAPT for 6 months and then aspirin as lifelong therapy [15,16]. However, patients involved in these studies had not high bleeding risk and no contraindications to anticoagulation.

Other studies suggest the safety of double antiplatelet therapy early in the first period after the procedure without warfarin transition, especially for patients with absolute contraindication to anticoagulation. The ASAP study (multicenter, non-randomized, including 150 patients implanted with a Watchman device) showed the feasibility of this approach, with 1 to 6 months of DAPT followed by aspirin lifelong [17]. Similarly, a recent analysis from the EWOLUTION registry (including 1005 patients successfully implanted with the Watchman device and with relative or absolute contraindication to oral anticoagulation) showed that, in a real-world population, both DAPT and NOAC are a safe alternative to warfarin after implantation [18].

On the other hand, antiplatelet drugs are more commonly prescribed rather than anticoagulants in studies involving Amplatzer devices implantation. Moreover, a recent study of Urena and colleagues evaluated 51 patients who underwent LAA occlusion with the AMPLATZER Cardiac Plug due to absolute contraindication to anticoagulant therapy. The procedure was followed by either DAPT or SAPT and a low rate of embolic and bleeding events was observed after a mean follow-up of 20 months [19].

To minimize bleeding events, there is an increasing interest in the possibility to use a “minimal” antithrombotic regimen after device implantation. One pilot study from Rodés-Cabau et al. including 31 patients with non-valvular AF, HAS-BLED > 3, CHA_2_DS_2_-VASc > 2, showed the preliminary safety and efficacy of SAPT (with either ASS or clopidogrel) following LAA occlusion with different devices [20].

Another single-center, prospective, non-randomized study on LAA occlusion with the ACP or Amulet device in a consecutive cohort of 110 patients, showed safety of ASS monotherapy after implantation without an increased risk of device-related thrombosis or stroke [21].

Our observational study seems to confirm these preliminary findings in a real-world cohort of patients with high bleeding and ischemic risk.

Even on a heterogeneous population, our study showed no difference between more standardized therapies (either anticoagulant or DAPT) and a “minimal” antithrombotic regimen prescribed at discharge soon after percutaneous LAA occlusion in terms of event-free survival rate (including silent left atrial thrombi, TIA, ischemic stroke, intracranial bleeding, other major and minor bleedings, mortality, and cardiovascular mortality) on both short and long-term FU.

These early findings support the hypothesis that patients with high bleeding risk (calculated by the HAS-BLED score) can benefit the most from a “minimal” drug regimen, with a reduced risk of bleeding events and without any apparent increase of embolic risk (considering both clinically relevant events and silent device or atrial thrombosis).

The main finding of our study is the low incidence of adverse events (both ischemic and hemorrhagic) after LAA occlusion.

In this setting, we can speculate that a tailored approach to antithrombotic therapy after the procedure, spacing from anticoagulant therapy to a “minimal” regimen with only ASS, seem to be a reasonable solution to reduce hemorrhagic complications, especially early after LAA occlusion.

The preliminary results of our experience, that must be confirmed by larger studies, seem to suggest that a “minimal” drug regimen is feasible without losing efficacy on embolic prevention for frailer categories of patients, with high bleeding risk.

In the absence of randomized clinical trials directly comparing therapeutic options, a tailored therapeutic regimen may be considered: there is still a rationale for prescribing OAC (with both warfarin or direct anticoagulants) or DAPT in patients with lower hemorrhagic risk, both early after LAA occlusion (to minimize embolic risk during device endothelialization) and long term (as LAA is not the only source of thrombo-embolic risk).

On the other hand, a less aggressive antithrombotic regimen with only one antiplatelet drug seems to be feasible and safe for patients with higher bleeding risk or with a previous major bleeding, thus preserving efficacy on stroke prevention and minimizing bleeding risk.

The main limitations of our research are reported below:

1. First of all, the relatively small sample size and the retrospective nature of the study do not allow these data to be generalized and can only support a hypothesis generating role of the present analysis.
2. Being an observational study, the choice of antithrombotic therapy was not based on a randomization but was left to the operator’s preference and usually based on ischemic and hemorrhagic risk evaluation. This led to a different distribution of patients in each group, with some differences in baseline characteristics. Our study enrolled a real-world population, which could however lead to some biases, such as missing follow-up data.
3. Different LAA occlusion devices were implanted in our center. While no data support the necessity for different antithrombotic regimens, we must underline that evidences obtained from one device may not be necessarily applicable to others.

## Figures and Tables

**Table 1:** Baseline characteristics. NIDDM: non-insulin dependent diabetes mellitus; IDDM: insulin dependent diabetes mellitus; TIA: transient ischemic attack; INR: international normalized ratio. GROUP A dual antiplatelet therapy; GROUP B anticoagulant therapy; GROUP C single antiplatelet therapy or no therapy; GROUP D combination of antiplatelet and anticoagulant therapy.

	OVERALL (*n* 260)	GROUP A (*n* 186)	GROUP B (*n* 50)	GROUP C (*n* 12)	GROUP D (*n* 12)	*p* Value
Age, Mean ± SD	72.7 ± 8.8	73 ± 8	73 ± 8	74 ± 9	72 ± 14	0.938
Male, *n* (%)	175 (67%)	128 (69%)	30 (60%)	7 (58%)	10 (83%)	0.355
NIDDM, *n* (%)	53 (20%)	34 (18.3%)	11 (22%)	6 (50%)	2 (16.7%)	0.117
IDDM, *n* (%)	22 (8.5%)	18 (9.7%)	3 (6%)	1 (8.3%)	0	0.603
Hypertension, *n* (%)	202 (77.7%)	153 (82%)	30 (60%)	9 (75%)	10 (83%)	0.157
Previous TIA, *n* (%)	24 (9.2%)	17 (9.1%)	5 (10%)	0	2 (16.7%)	0.389
Previous Stroke, *n* (%)	66 (25.4%)	44 (23.7%)	12 (24%)	6 (50%)	4 (33.3%)	0.255
Previous Major Bleeding, *n* (%)	147 (56.5%)	103 (55.4%)	31 (62%)	8 (66.7%)	5 (41.7%)	0.514
Congestive Heart Failure, *n* (%)	74 (28.5%)	60 (32%)	10 (20%)	1 (8.3%)	3 (35%)	0.137
Coronary artery disease patients, *n* (%)	84 (32%)	71 (38%)	4 (8%)	2 (17%)	7 (58%)	0.005
Ejection Fraction, Mean ± SD	52 ± 10	51 ± 10	54 ± 9	58 ± 3	53 ± 9	0.05
Creatinine Clearance (mL/min), Mean ± SD	59 ± 27	56 ± 27	68 ± 29	59 ± 32	64 ± 27	0.07
Chronic Kidney Disease, *n* (%)	83 (31.9%)	64 (34.4%)	11 (22%)	4 (33.3%)	4 (33.3%)	0.593
Dialysis, *n* (%)	17 (6.5%)	14 (7.5%)	1 (2%)	2 (16.7%)	0	0.483
Hepatic Failure, *n* (%)	13 (5%)	9 (4.8%)	2 (4%)	2 (17%)	0	0.314
Labile INR, *n* (%)	27 (10.4%)	23 (12.4%)	2 (4%)	0	2 (16.7%)	0.190
CHA_2_DS_2_-VASc score, Mean ± SD	3.8 ± 1.7	3.9 ± 1.7	3.3 ± 1.7	4.5 ± 1.8	4 ± 1.5	0.102
HAS-BLED score, Mean ± SD	3.6 ± 1.4	3.7 ± 1.4	3 ± 1.3	4.2 ± 1.3	3.9 ± 1.4	0.005

**Table 2:** Procedural data. GROUP A dual antiplatelet therapy; GROUP B anticoagulant therapy; GROUP C single antiplatelet therapy or no therapy; GROUP D combination of antiplatelet and anticoagulant therapy.

	OVERALL (*n* 260)	GROUP A (*n* 186)	GROUP B (*n* 50)	GROUP C (*n* 12)	GROUP D (*n* 12)	*p* Value
Devices implanted, *n* (%): Watchman, Watchman FLX Amplatzer Amulet	82 (31%)	57 (30%)	19 (38%)	3 (25%)	3 (25%)	0.641
	5 (2%)	1 (0.5%)	4 (8%)	0	0	0.028
	55 (21%)	44 (23%)	2 (4%)	5 (42%)	4 (33%)	0.002
	118 (45%)	84 (45%)	25 (50%)	4 (33%)	5 (42%)	0.709
Size Change, *n* (%)	5 (2%)	4 (2.1%)	0	0	1 (8.3%)	0.312
Repositioning, *n* (%)	14 (5.4%)	10 (5.4%)	1 (2%)	2 (16.7%)	1 (8.3%)	0.235
Procedural success, *n* (%)	260 (100%)	186 (100%)	50 (100%)	12 (100%)	12 (100%)	N/A

**Table 3:** Clinical follow up at three months. TIA: transient ischemic attack; CV: cardiovascular. GROUP A dual antiplatelet therapy; GROUP B anticoagulant therapy; GROUP C single antiplatelet therapy or no therapy; GROUP D combination of antiplatelet and anticoagulant therapy.

Events, *n* (%)	OVERALL (*n* 260)	GROUP A (*n* 186)	GROUP B (*n* 50)	GROUP C (*n* 12)	GROUP D (*n* 12)	*p* Value
Device-related thrombosis	3 (1.2%)	2 (1.1%)	1 (2.1%)	0	0	0.892
Ischemic Stroke	1 (0.4%)	0	1 (2.1%)	0	0	0.232
TIA	1 (0.4%)	0	0	0	1 (9.1%)	0.005
Stroke and TIA	2 (0.8%)	0	1 (2.1%)	0	1 (9.1%)	0.07
Intracranial Bleeding	2 (0.8%)	0	2 (4.2%)	0	0	0.08
Major Bleeding	5 (2%)	2 (1.1%)	1 (2.1%)	1 (8.3%)	1 (9.1%)	0.107
Minor Bleeding	10 (4%)	7 (3.8%)	3 (6.3%)	0	0	0.655
Mortality	1 (0.4%)	1 (0.5%)	0	0	0	0.942
CV Mortality	1 (0.4%)	1 (0.5%)	0	0	0	0.942

**Table 4:** Long term clinical follow-up. FU: follow-up; TEE: transesophageal echocardiography; TIA: transient ischemic attack. GROUP A dual antiplatelet therapy; GROUP B anticoagulant therapy; GROUP C single antiplatelet therapy or no therapy; GROUP D combination of antiplatelet and anticoagulant therapy.

	OVERALL (*n* 260)	GROUP A (*n* 186)	GROUP B (*n* 50)	GROUP C (*n* 12)	GROUP D (*n* 12)	*p* Value
Clinical FU, *n* (%)	253 (97.3%)	182 (97.8%)	48 (96%)	12 (100%)	11 (91.7%)	0.508
Clinical FU (days) Median (IQR)	420 (250–704)	434 (265–704)	382 (197–715)	554 (255–904)	347 (188–595)	0.706
3 months TEE FU, *n* (%)	194 (74.6%)	148 (79.6%)	32 (64%)	8(66.7%)	6(50%)	0.022
TEE data, n (%):						
leaks (any)	41 (21%)	30 (20%)	8 (24%)	1 (2.4%)	2 (4.9%)	0.753
major leaks (>5 mm)	3 (1.1%)	1 (0.5%)	1 (2%)	1 (8.3%)	0	0.090
device related thrombosis	4 (1.6%)	2 (1%)	1 (2%)	0	1 (8.3%)	0.092
Events, n (%):						
Ischemic Stroke	5 (2%)	3 (1.6%)	2 (4%)	0	0	0.926
TIA	3 (1.2%)	0	1 (2%)	0	2 (16.6%)	0.005
Stroke and TIA	8 (3.1%)	3 (1.6%)	3 (6%)	0	2 (16.6%)	0.014
Intracranial Bleeding	3 (1.2%)	1 (0.5%)	2 (4%)	0	0	0.217
Major Bleeding	7 (2.7%)	3 (1.6%)	1 (2%)	2 (16.6%)	1 (8.3%)	0.016
Minor Bleeding	13 (5%)	10 (5.4%)	3 (6%)	0	0	0.639
Mortality	15 (5.7%)	12 (6.5%)	0	0	3 (25%)	0.059
CV Mortality	8 (3.1%)	7 (3.8%)	0	0	1 (8.3%)	0.631

The fields “Stroke and TIA”, “Major Bleeding” and “Mortality” respectively report the cumulative incidence in the overall population and in the patients belonging to each group of: stroke and TIA, intracranial bleeding and major bleeding of other districts, CV mortality and mortality due to other reasons.

## Data Availability

Data sharing is available following a written request addressed to dangelo.giuseppe@hsr.it.

## Supplementary Materials

The following are available online at https://www.mdpi.com/article/10.3390/jcm10091959/s1, Table S1: Baseline characteristics of the general population and of the 40 patients not included in the final analysis due to exclusion criteria.

## References

[1] Wolf P.A., Abbott R.D., Kannel W.B. (1991). Atrial fibrillation as an independent risk factor for stroke: The Framingham Study. Stroke.

[2] Lin H.J., Wolf P.A., Kelly-Hayes M., Beiser A.S., Kase C.S., Benjamin E.J., D’Agostino R.B. (1996). Stroke severity in atrial fibrillation. The Framingham Study. Stroke.

[3] Hart R.G., Pearce L.A., Aguilar M.I. (2007). Meta-analysis: Antithrombotic therapy to prevent stroke in patients who have nonvalvular atrial fibrillation. Ann. Intern. Med..

[4] Connolly S.J., Ezekowitz M.D., Yusuf S., Eikelboom J., Oldgren J., Parekh A., Pogue J., Reilly P.A., Themeles E., Varrone J. (2009). RE-LY Steering Committee and Investigators. Dabigatran versus warfarin in patients with atrial fibrillation. N. Engl. J. Med..

[5] Patel M.R., Mahaffey K.W., Garg J., Pan G., Singer D.E., Hacke W., Breithardt G., Halperin J.L., Hankey G.J., Piccini J.P. (2011). ROCKET AF Investigators. Rivaroxaban versus warfarin in nonvalvular atrial fibrillation. N. Engl. J. Med..

[6] Granger C.B., Alexander J.H., McMurray J.J., Lopes R.D., Hylek E.M., Hanna M., Al-Khalidi H.R., Ansell J., Atar D., Avezum A. (2011). ARISTOTLE Committees and Investigators. Apixaban versus warfarin in patients with atrial fibrillation. N. Engl. J. Med..

[7] Healey J.S., Crystal E., Lamy A., Teoh K., Semelhago L., Hohnloser S.H., Cybulsky I., Abouzahr L., Sawchuck C., Carroll S. (2005). Left Atrial Appendage Occlusion Study (LAAOS): Results of a randomized controlled pilot study of left atrial appendage occlusion during coronary bypass surgery in patients at risk for stroke. Am. Heart J..

[8] Ostermayer S.H., Reisman M., Kramer P.H., Matthews R.V., Gray W.A., Block P.C., Omran H., Bartorelli A.L., Della Bella P., Di Mario C. (2005). Percutaneous left atrial appendage transcatheter occlusion (PLAATO system) to prevent stroke in high-risk patients with non-rheumatic atrial fibrillation: Results from the international multi-center feasibility trials. J. Am. Coll. Cardiol..

[9] Holmes D.R., Reddy V.Y., Turi Z.G. (2009). Percutaneous closure of the left atrial appendage versus warfarin therapy for prevention of stroke in patients with atrial fibrillation: A randomised non-inferiority trial. Lancet.

[10] Syed F.F., DeSimone C.V., Friedman P.A., Asirvatham S.J. (2014). Left atrial appendage exclusion for atrial fibrillation. Cardiol. Clin..

[11] Akin I., Nienaber C.A. (2017). Left atrial appendage occlusion: A better alternative to anticoagulation?. World J. Cardiol..

[12] Phillips K.P., Paul V. Dealing with the Left Atrial Appendage for Stroke Prevention: Devices and Decision-Making. Heart Lung Circ..

[13] Leon M.B., Piazza N., Nikolsky E., Blackstone E.H., Cutlip D.E., Kappetein A.P., Krucoff M.W., Mack M., Mehran R., Miller C. (2011). Standardized endpoint definitions for Transcatheter Aortic Valve Implantation clinical trials: A consensus report from the Valve Academic Research Consortium. J. Am. Coll. Cardiol..

[14] Camm A.J., Lip G.Y., De Caterina R., Savelieva I., Atar D., Hohnloser S.H., Hindricks G., Kirchhof P. (2012). ESC Committee for Practice Guidelines (CPG). 2012 focused update of the ESC Guidelines for the management of atrial fibrillation: An update of the 2010 ESC guidelines for the management of atrial fibrillation. Developed with the special contribution of the European Heart Rhythm Association. Eur. Heart J..

[15] Reddy V.Y., Holmes D., Doshi S.K., Neuzil P., Kar S. (2011). Safety of percutaneous left atrial appendage closure: Results from the Watchman Left Atrial Appendage System for Embolic Protection in Patients with AF (PROTECT AF) clinical trial and the Continued Access Registry. Circulation.

[16] Holmes D.R., Kar S., Price M.J., Whisenant B., Sievert H., Doshi S.K., Huber K., Reddy V.Y. (2014). Prospective randomized evaluation of the Watchman Left Atrial Appendage Closure device in patients with atrial fibrillation versus long-term warfarin therapy: The PREVAIL trial. J. Am. Coll. Cardiol..

[17] Reddy V.Y., Möbius-Winkler S., Miller M.A., Neuzil P., Schuler G., Wiebe J., Sick P., Sievert H. (2013). Left atrial appendage closure with the Watchman device in patients with a contraindication for oral anticoagulation: The ASAP study (ASA Plavix Feasibility Study With Watchman Left Atrial Appendage Closure Technology). J. Am. Coll. Cardiol..

[18] Bergmann M.W., Betts T.R., Sievert H., Schmidt B., Pokushalov E., Kische S., Schmitz T., Meincke F., Stein K.M., Boersma L.V. (2017). Early Anticoagulation drug regimens after WATCHMAN Left Atrial Appendage Closure: Safety and efficacy. EuroIntervention.

[19] Urena M., Rodés-Cabau J., Freixa X., Saw J., Webb J.G., Freeman M., Horlick E., Osten M., Chan A., Marquis J.F. (2013). Percutaneous left atrial appendage closure with the AMPLATZER cardiac plug device in patients with nonvalvular atrial fibrillation and contraindications to anticoagulation therapy. J. Am. Coll. Cardiol..

[20] Rodriguez-Gabella T., Nombela-Franco L., Regueiro A., Jiménez-Quevedo P., Champagne J., O’Hara G., Bernier M., Macaya C., Rodés-Cabau J. (2016). Single Antiplatelet Therapy Following Left Atrial Appendage Closure in Patients with Contraindication to Anticoagulation. J. Am. Coll. Cardiol..

[21] Korsholm K., Nielsen K.M., Jensen J.M., Jensen H.K., Andersen G., Nielsen-Kudsk J.E. (2017). Transcatheter left atrial appendage occlusion in patients with atrial fibrillation and a high bleeding risk using aspirin alone for post-implant antithrombotic therapy. EuroIntervention.

