# Supplementary material for: Role of Different Antithrombotic Regimens after Percutaneous Left Atrial Appendage Occlusion: A Large Single Center Experience
Source: J Clin Med. 2021 May 2;10(9):1959. doi: 10.3390/jcm10091959 (PMC8124741; doi:10.3390/jcm10091959)
Supplement: Supplementary file 1 [file jcm-10-01959-s001.zip › jcm-1158220-supplementary.pdf]

Table S1. Baseline characteristics of the general population and of the 40 patients not included in the final analysis due to exclusion criteria.

.	**OVERALL (*n* 260)**	**EXCLUDED (*n* 40)**
Age, Mean ± SD	72.7 ± 8.8	72.7 ± 11.3
Male, *n* (%)	175 (67%)	16 (40%)
NIDDM, *n* (%)	53 (20%)	4 (10%)
IDDM, *n* (%)	22 (8.5%)	1 (2.5%)
Hypertension, *n* (%)	202 (77.7%)	21 (52.5%)
Previous TIA, *n* (%)	24 (9.2%)	3 (7.5%)
Previous Stroke, *n* (%)	66 (25.4%)	10 (25%)
Previous Major Bleeding, *n* (%)	147 (56.5%)	16 (40%)
Congestive Heart Failure, *n* (%)	74 (28.5%)	16 (40%)
Coronary artery disease patients, *n* (%)	84 (32%)	12 (30%)
Ejection Fraction, Mean ± SD	52 ± 10	62.5 ± 13.6
Creatinine Clearance (ml/min), Mean ± SD	59 ± 27	58.1 ± 26.1
Chronic Kidney Disease, *n* (%)	83 (31.9%)	12 (30%)
Dialysis, *n* (%)	17(6.5%)	1 (2.5%)
Hepatic Failure, *n* (%)	13 (5%)	0
Labile INR, *n* (%)	27 (10.4%)	6 (15%)
CHA_2DS_2-VASc score, Mean ± SD	3.8 ± 1.7	4.1 ± 1.6
HAS-BLED score, Mean ± SD	3.6 ± 1.4	3.4 ± 1.4
Previous trans-catheter ablation, *n* (%)	42 (16.2%)	2 (5%)
Previous surgical ablation, *n* (%)	1 (0.4%)	1 (2.5%)

NIDDM: non-insulin dependent diabetes mellitus; IDDM: insulin dependent diabetes mellitus; TIA: transient ischemic attack; INR: international normalized ratio.
